# Dual-point sensory anchoring: emergent attentional coherence through embodied coupling

**DOI:** 10.3389/fnins.2026.1887791

**Published:** 2026-09-25

**Authors:** Phillip Fei Hou

**Affiliations:** Independent Researcher, London, United Kingdom

**Keywords:** active inference, attentional stability, cognitive resilience, embodied cognition, precision weighting, predictive processing, sensory gating, somatosensory perception

## Abstract

Attentional instability is a persistent cognitive challenge in adult life, yet most existing interventions work through top-down regulatory pathways that require sustained effort, specialized instruction, or clinical access. This paper introduces Dual-Point Sensory Anchoring (DPSA), a sensorimotor paradigm proposing a different route: attentional stability engineered from the bottom up, through the continuous confirmation of low-level tactile-proprioceptive predictions instead of through metacognitive monitoring. The paradigm is organized around four interacting constraints: simultaneous bilateral whole-body movement, deliberate gating of visual and auditory input, multi-dimensional fingertip anchoring as the primary locus of sensory prediction confirmation, and uninterrupted sensorimotor continuity. No existing paradigm, to our knowledge, assembles all four. Within the predictive processing and active inference framework, this configuration is predicted to confine prediction-error resolution preferentially to fast, short-latency somatosensory and cerebellar circuits, producing a low-entropy attentional regime that deepens across the session. Low executive demand is a predicted and empirically testable property of the configuration, not a definitional one. Eleven directional falsifiable predictions are set out, with six control conditions in two families, most of them testable with standard laboratory equipment. DPSA is presented as an as-yet unvalidated theoretical model: no empirical data are reported, and none of its mechanistic claims have yet been tested. The framework addresses a translational need for low-burden, theoretically grounded mind-body interventions supporting cognitive vitality across diverse populations and settings.

## 1 Introduction

Attentional instability — the tendency of the mind to drift, to fragment and to resist sustained engagement — is among the most pervasive cognitive challenges of contemporary life. Its consequences are not merely subjective: elevated attentional volatility is associated with increased default mode network activity and with measurable costs to cognitive performance (Smallwood and Schooler, 2015; Seli et al., 2018). Commonly used interventions, including meditation, pharmacological approaches and cognitive training, address the problem through top-down regulatory pathways and require sustained effort, specialized instruction or clinical access. The embodied cognition framework suggests another approach. If cognitive processes arise from dynamic interactions between brain, body and environment, then designing the sensorimotor environment deliberately may offer a bottom-up route to attentional stability, one that asks much less of metacognitive regulation. That possibility has not yet been specified in enough experimental detail to be investigated directly. The key, this paper proposes, lies in the architecture of somatosensory perception itself: the continuous, bilateral and differentiable tactile-proprioceptive signals generated by structured whole-body movement constitute a perceptual environment whose prediction-confirming properties can be experimentally engineered with precision. Dual-Point Sensory Anchoring (DPSA) is offered as that specification.

This paper introduces and theoretically grounds the DPSA framework as a direct response to that open question. It is organized in five parts. First, we review the theoretical traditions that bear on attentional stability during embodied movement and what each contributes to DPSA’s question. Second, we identify the open experimental question that these traditions collectively raise, and that no existing paradigm has been designed to answer. Third, we present the DPSA framework itself, developing the mechanistic and computational account of how bilateral movement structure is proposed to produce attentional stability. Fourth, we describe the canonical DPSA protocol, the experimental procedure through which the framework is operationalized and can be replicated. Fifth, we set out the empirical predictions the framework generates, the control conditions that could falsify it, and the translational implications for future research.

We begin with the theoretical landscape from which DPSA emerges: the established frameworks that have each approached this problem, and the consistent gap their convergence reveals.

## 2 State of the art

The question of how the brain achieves and maintains attentional stability during embodied movement has been approached from several independent theoretical traditions. None was designed to answer DPSA’s particular question. Each, however, has identified properties that bear on it.

The most formally developed of these accounts starts from the brain’s fundamental organizing principle. Contemporary neuroscience increasingly interprets perception, action and attention as expressions of a single integrative process: the continuous minimization of prediction error within hierarchical generative models of the world (Rao and Ballard, 1999; Friston, 2010; Clark, 2013). Perception, on this view, is active inference rather than passive reception; the brain constructs its best hypothesis about the causes of sensory input and revises it when prediction errors demand. Attention is treated as a precision-weighting mechanism, the selective amplification of reliable sensory evidence relative to noise, not as a spotlight directed by will (Feldman and Friston, 2010). Active inference extends the framework to action, proposing that organisms move in ways that generate the sensory inputs they expect (Friston et al., 2016; Parr et al., 2022). From this tradition DPSA takes its computational vocabulary: precision weighting, hierarchical inference, prediction error minimization. These are the terms in which its proposed mechanisms can be formally specified and empirically tested. DPSA is a proposal about how to engineer the perceptual environment so that the brain’s predictive architecture stabilizes attention from the bottom up.

Motor control and cerebellar forward-model frameworks establish that smooth, predictable, well-practiced movement reduces corrective demand and lowers the cost of motor coordination (Todorov and Jordan, 2002; Scott, 2004; Wolpert and Kawato, 1998; Wolpert et al., 2011). A forward model is the brain’s internal simulation of what a movement will feel like before it happens. When the simulation matches the actual sensory outcome, correction is minimal and movement proceeds automatically, without conscious attention. This work is empirically robust and bears directly on continuous skilled movement. It provides the foundation on which DPSA builds its account of how movement-level stability may propagate into attentional and autonomic circuits.

Dynamical systems approaches show that stable coordination patterns can emerge spontaneously from self-organizing coupled oscillators, without centralized control (Kelso, 1995; Tognoli and Kelso, 2014; Haken et al., 1985). Bimanual coordination, phase transitions and metastability have been systematically characterized within this framework, showing that bilateral movement structure, rather than cognitive effort, can itself serve as a primary source of stability in neural and motor systems. Applied to DPSA, this leads to the prediction that its slow bilateral whole-body movement may find and sustain a stable coordination pattern through the physics of the body itself, without the participant having to maintain it deliberately.

Ecological psychology identifies the sensory boundary as the primary site of perceptual organization, with perception and action always embedded in a meaningful coupling between organism and world (Gibson, 1979; Turvey, 1992). This is what motivates DPSA’s treatment of the fingertip anchors as the primary locus of sensory prediction confirmation. The precise design of the contact point between organism and sensory environment is what selects the fingertip anchors as the proposed sensory boundary at which tactile-proprioceptive predictions are continuously tested and confirmed.

Embodied and enactive cognition frameworks hold that stable perception is grounded in the predictability and lawfulness of sensorimotor contingencies. Perceptual stability arises through active sensorimotor engagement, not passive sensory reception (O’Regan and Noë, 2001; Noë, 2004; Varela et al., 1991; Thompson et al., 2017). Knowing where one’s hand is in space, for instance, requires continuous active touch: the sensory consequence of movement confirms the prediction, and stability follows from that confirmation rather than from internal representation alone. This directly supports DPSA’s proposal that a continuously confirming sensorimotor environment may sustain attentional stability with low executive demand, in place of deliberate cognitive regulation.

Contemplative and attentional practice research indicates that attentional stability is neurally grounded and trainable, and that the cost of unconstrained attention is measurable (Lutz et al., 2008; Tang et al., 2015; Smallwood and Schooler, 2015; Seli et al., 2018). This lends empirical support to DPSA’s proposal that a low-cognitive-load route to attentional stability is both theoretically plausible and practically valuable.

Research on large-scale brain network dynamics establishes that attentional instability has a measurable neural signature: dominance of the default mode network (DMN; Raichle et al., 2001; Buckner et al., 2008). The DMN is a set of midline cortical regions whose activity is inversely related to externally directed attention, and whose engagement correlates with mind-wandering, elevated autonomic arousal and reduced cognitive performance (Smallwood and Schooler, 2015). Sustained attentional engagement is associated with suppression of DMN activity and coordinated recruitment of the frontoparietal attention and salience networks (Fox et al., 2005; Menon and Uddin, 2010). Maintained through top-down regulation alone, that reorganization is metabolically costly. Structured sensorimotor engagement, however, can alter the same configuration without deliberate cognitive effort (Spreng and Schacter, 2012; Deco et al., 2011), which means bottom-up sensory input may shift network state through mechanisms that do not depend on executive control. This provides direct network-level grounding for DPSA’s central proposal: that a precisely engineered sensorimotor environment may produce the large-scale network shift associated with attentional stability from the bottom up, and may do so with lower executive demand than top-down regulation requires.

Bilateral stimulation paradigms confirm that bilateral sensory organization is a neurologically relevant modulator of cognitive and physiological state (Shapiro, 2001; van den Hout and Engelhard, 2012). Eye Movement Desensitization and Reprocessing (EMDR) employs alternating bilateral sensory input for therapeutic purposes, which establishes it as a clinically studied principle. Its relevance to DPSA’s simultaneous, self-generated fingertip input is analogical, and the simultaneous configuration requires empirical testing as the central architectural feature of the paradigm. DPSA departs from EMDR in using simultaneous rather than sequential bilateral activation, and the departure is theoretically motivated: simultaneous engagement is proposed to establish a symmetric bilateral precision landscape in place of a temporally alternating one, and it is the symmetric configuration that the hemispheric coherence account developed below requires.

These eight traditions reached their conclusions by methods as varied as mathematical modeling, neurophysiology, philosophical analysis, clinical observation, ecological fieldwork, contemplative research and large-scale network neuroimaging. Each has identified, from a different direction, properties that bear on the question DPSA asks: bilateral symmetry, continuous low-jerk movement, sensorimotor regularity, bottom-up stabilization of attentional and physiological states, and the sensory boundary as the primary locus of cognitive coherence. The convergence is what matters here. Separate routes arriving at the same design properties suggest that those properties reflect recurring organizational principles in biological systems, and it is those principles that DPSA is designed to exploit experimentally by assembling them within a single controlled paradigm.

## 3 The consistent gap

The convergence described above raises a precise experimental question. Eight independent traditions, developed through different methods, have identified overlapping design properties relevant to attentional stability during embodied movement. No single paradigm has assembled those properties simultaneously within a single controlled experimental framework. Without that assembly, how they interact to shape hierarchical inference cannot be investigated empirically.

The problem is neither a lack of evidence nor a lack of theoretical attention. Each tradition has addressed the problem it was designed to solve, and has done so with substantial empirical support. The gap is one of experimental specification. No existing paradigm defines, with enough precision for replication, how bilateral symmetry, continuous movement geometry, uninterrupted sensorimotor continuity, deliberate sensory gating and multi-dimensional attentional anchoring combine to shape the inferential and energetic conditions under which attentional stability arises. Taken together, these traditions point toward a common unresolved experimental question, one that falls outside the scope of any single tradition precisely because answering it requires their simultaneous instantiation.

That is the question DPSA is designed to answer. It replaces none of the frameworks described above. It supplies the experimental instantiation their convergence points toward: a specified, reproducible behavioral paradigm that assembles these properties, creating the conditions under which their combined effect on hierarchical inference can be observed and measured.

Dual-Point Sensory Anchoring is designed around four defining constraints, and together they constitute its novelty and its experimental contribution. The first is simultaneous bilateral whole-body movement: both hands trace coordinated elliptical paths with the body’s weight shifting in phase, engaging bilateral somatosensory and motor circuits at the same time rather than in alternation or in sequence. The second is deliberate sensory gating: eyes closed and auditory input attenuated, conditions engineered to elevate the relative precision of tactile and proprioceptive channels and to reduce cross-modal competition. The third is multi-dimensional fingertip anchoring. Attention is directed to the anchors as combined spatial, kinematic, tactile and environmental reference points, providing a continuously updating sensory stream that resists adaptation and sustains engagement without effort.

The fourth is uninterrupted sensorimotor continuity. Once the anchoring phase is established, successive revolutions of the trajectory contain no task-defined terminal target, no pause, no reversal of direction and no reset: the final segment of one revolution passes directly into the next, and the direction of travel is held constant within a session. The constraint is defined by these properties of the protocol and makes no claim about the participant’s attention. Two forms of closure are involved and should be distinguished. Geometric closure means that the path returns upon itself; feedback closure means that the sensory consequences of movement continuously inform the movement that follows. Smooth differentiability of the trajectory is an implementation feature supporting this continuity, not part of the constraint itself. The hypothesis that follows — and it is a hypothesis and not a definition — is that an unbroken trajectory of this kind removes the recurrent boundaries at which attention would otherwise be reoriented, so that the attentional relation to the anchors can be sustained while their sensory content changes continuously.

The four constraints answer different questions. Bilateral whole-body movement describes what the body does and the postural coupling through which it is realized. Sensory gating describes the sensory environment. Fingertip anchoring describes where attention is organized. Uninterrupted sensorimotor continuity describes how the relation among them is preserved through time; it is a temporal constraint, not a further property of the movement. DPSA is characterized as guided somatosensory attention with hypothesized low executive demand. Attending to the anchors, maintaining posture during weight transfer, and drawing on previously acquired coordination all require attentional control. Executive demand is therefore treated as an empirical variable, indexed by perceived-effort and attentional-engagement ratings and by frontal theta.

Existing practices have each developed around different purposes and different mechanisms. Meditation cultivates attentional focus through metacognitive monitoring, which is a different regulatory route from DPSA’s bottom-up sensory architecture. Tai Chi and Qigong share DPSA’s bilateral embodied character but differ from it in what they specify. Tai Chi is performed as a sequence of discrete movements: continuity holds within a movement, but each terminates at a defined posture and the sensorimotor relation is reset before the next begins. Each movement has a target, and the instruction directs attention toward reaching it. The perceptual content of the path traveled is not what the practice asks the practitioner to attend to, whatever an individual may register. DPSA specifies the reverse. Its landmark positions are descriptive markers, not targets, and the evolving sensory content between them is the object of attention throughout.

Qigong covers a wider range of practices, some involving no movement at all, and is organized around regulation of the breath and a calm state directed toward relaxation. Both are conditions DPSA removes rather than specifies: respiration is left unregulated, and relaxation is treated as an alternative explanation that the control conditions exist to exclude. Where attention is anchored in Qigong, it is anchored to interoceptive sensation, to the breath or to internal bodily state, and not to an external tactile boundary.

Eye Movement Desensitization and Reprocessing employs bilateral sensory organization for therapeutic purposes through alternating input. Each of these approaches is valuable within its own domain. We are not aware of any existing paradigm that engineers all four of DPSA’s defining constraints simultaneously within a controlled experimental design, and it is the combination, rather than any single element, that constitutes DPSA’s theoretical and experimental contribution.

The comparison below sets DPSA against the embodied and contemplative practices with which it shares features. Entries characterize what each practice specifies structurally, not what it achieves empirically; several of these practices have substantial evidence bases that are not at issue here. The point of the table is narrower: each shares one or more of DPSA’s constraints, and none specifies all four. In the final column, a practice is scored Yes only where recurrent movement proceeds without instructed pauses, reversals, target reacquisition or inter-cycle reset during the relevant phase; Varies indicates that the property is not constitutive of the practice and differs across forms or teachers. Individual schools or implementations may differ from the entry shown. Novelty is claimed for the conjunction of the four constraints, never for continuous movement alone (Table 1).

**TABLE 1:** Dual-Point Sensory Anchoring (DPSA) and related practices compared against the four defining constraints.

Practice	Bilateral whole-body movement	Sensory gating	Fingertip anchoring	Uninterrupted sensorimotor continuity
DPSA	Yes	Yes — eyes closed, controlled auditory masking	Yes — primary locus	Yes — no pause, reversal or reset
Tai Chi	Yes	No — eyes open, form-following	No — attention on form and on reaching each posture	No — sequence of discrete movements, each ending at a defined posture with a reset before the next
Qigong	Varies — movement is not constitutive; some forms are static	No — breath regulation adds a channel rather than reducing competing ones	No — attention anchored to breath or internal state, not an external tactile boundary	Varies — differs across forms
Feldenkrais (Awareness Through Movement)	Partial — often unilateral or segmental	No — verbal instruction throughout	No	Varies — many lessons include deliberate pauses and returns to rest
Mindfulness-based movement (e.g., MBSR)	Partial	No — eyes may be open; verbal guidance	No — attention on breath or body scan	Varies — not constitutive of the practice
Walking meditation	Partial — gait is bilateral but alternating	No — eyes open for navigation	No — attention on foot contact or breath	Varies — turns and pauses at path ends are common
Focused-attention meditation (breath)	No — stationary	Partial — eyes closed, no movement	No — interoceptive locus	No — stationary
Bilateral stimulation (EMDR protocols)	No — stimulation applied, not self-generated	No	No — externally delivered, not attended as anchor	No — alternating, with directional reversal
Mudra practice	No — static hand posture	Partial	Partial — hand-focused but static	No — static posture

Subsequent sections develop this contribution in full. Section 4 presents the mechanistic and computational account of how the four constraints interact to produce what is termed here a low-entropy attentional regime: a theoretical description of a progressively constrained distribution of sensorimotor states, indexed in practice by measures of variability and dynamical stability, not by information-theoretic entropy (Section 6). Section 5 describes the canonical protocol through which the framework is operationalized. Section 6 sets out the empirical predictions the framework generates and the conditions under which they can be falsified.

## 4 The DPSA framework

This section develops the mechanistic account in ten steps, moving outward from the tactile boundary through the computational architecture, the laminar circuitry, the energetic and oscillatory consequences, and finally into the autonomic and interoceptive hierarchy. Section 4.10 then sets out how much confidence each layer of the account can bear, before the experimental protocol in Section 5.

### 4.1 Tactile architecture and bilateral anchoring

The account begins at the tactile boundary. The fingertips are among the most densely innervated regions of the human body. Merkel and Ruffini endings encode static pressure and skin stretch; Meissner and Pacinian corpuscles signal dynamic shear and vibration (Johansson and Flanagan, 2009). Their Aβ afferents travel via the dorsal-column medial-lemniscus pathway to enlarged fingertip territories in primary somatosensory cortex, providing high-fidelity, continuously updating tactile evidence of exceptional spatial resolution. Those cortical territories contain more than contralateral representations alone. They can form bilateral receptive fields through posterior callosal fibers, and corresponding finger zones across both hands can respond synchronously to bilateral stimulation (Iwamura et al., 1994; Tamè et al., 2021). The single-unit evidence for this comes from macaque postcentral cortex; the human evidence is convergent but indirect.

Evidence from the rodent whisker system indicates that bilateral integration in primary somatosensory cortex is state-dependent: it emerges during goal-directed active touch and is mediated by callosal signaling (Park et al., 2025). This is a rodent finding in a different sensory modality. It motivates the present account by analogy and does not establish that the same mechanism operates in human fingertip touch. DPSA’s bilateral fingertip anchors are designed to engage the bilateral somatosensory architecture described above, providing two stable, synchronously active somatosensory channels through which attentional allocation is proposed to be distributed symmetrically across hemispheres with low executive demand.

The attentional structure that results from this bilateral engagement is not, we propose, adequately described as divided allocation between two fully independent foci. Evidence from the tactile modality points away from complete independence. In the passive-stimulation paradigm of Kusnir et al. (2024), stimulation at an unattended contralateral site influenced detection at a fixed attended site, and detection performance tracked the combined bilateral signal despite instructions to ignore the other site. The findings support obligatory cross-site interaction under those experimental conditions. They do not establish perceptual fusion, and they do not determine whether site-specific sensory content remains distinguishable. DPSA differs further in that its tactile input is continuously self-generated and may therefore be attenuated, through prediction of its sensory consequences, relative to externally applied stimulation. Whether comparable cross-site interaction occurs during DPSA is untested; the unilateral and movement-matched conditions begin to address it.

Dual-Point Sensory Anchoring proposes that this cross-site interaction may support a relational bilateral structure. On that account, attention is organized around the bilateral configuration and its moment-to-moment covariation, and does not alternate between two independently monitored targets. The relational information is genuinely bilateral because it cannot be specified by either anchor considered alone. Whether each anchor’s sensory content remains individually distinguishable is not assumed here and requires dedicated perceptual probing. Three consequences follow. The account needs no claim that two is an optimal number of attentional foci. It predicts that unilateral anchoring will be less effective, not because two targets are easier to sustain than one, but because a unilateral anchor does not establish the simultaneous symmetric bilateral configuration proposed here. And it makes interhemispheric coherence a predicted signature of bilateral relational processing rather than an additional assumption.

The available evidence therefore supports a plausible bilateral sensory architecture from which the relational-binding hypothesis can be developed. Section 4.2 sets out how those sensory conditions must be deliberately prepared.

### 4.2 Sensory gating and channel prioritization

Deliberate gating of visual and auditory input turns this bilateral foundation into a dominant sensory channel. Eye closure elevates occipital-parietal alpha power and reduces the relative gain of visual input, while controlled low-salience auditory masking reduces the intrusiveness and variability of competing auditory input; together these increase the relative weight of somatosensory evidence (Jensen and Mazaheri, 2010; Klimesch, 2012). Under these conditions, tactile and proprioceptive channels are proposed to assume priority. On the precision-weighting account set out above, reducing exteroceptive load is expected to enhance somatosensory processing. The gating is engineered, not incidental. With visual and auditory intrusion minimized, and in the absence of unstructured social cues, the bilateral fingertip anchors become the principal sensory inputs, and the thalamus regulates cortical gain accordingly, maintaining stable precision on both channels. The result is proposed to be a low-variance, somatosensory-dominant sensory field in which tactile and proprioceptive evidence may come to dominate the generative model through the architectural reduction of competing signal sources rather than through effortful suppression of other modalities. The eyes-closed, low-noise conditions of DPSA can therefore be read as precision engineering: visual alpha-mediated gating, together with a stable low-salience auditory environment, enhances somatosensory gain so that the fingertip anchors can function as the brain’s primary source of sensory evidence.

### 4.3 The necessity of continuous movement and whole-body coupling

Within this prepared sensory field, continuous movement is necessary. A static bilateral configuration might appear sufficient: sustained tactile contact should provide predictable bilateral input without the complexity of coordinated movement. Three considerations specific to the predictive processing account explain why continuous differentiable motion is theoretically preferable to static bilateral contact, namely sensory adaptation, postural drift and epistemic value. First, sensory adaptation. Slowly adapting type I Merkel afferents show response attenuation within seconds of constant stimulation, while rapidly adapting Meissner corpuscles cease firing entirely under static conditions (Gescheider et al., 1994; Johnson, 2001). Adaptation reduces the precision of the tactile channel, allowing other modalities or internal mentation to capture attentional resources at exactly the point where stability is most needed. Continuous movement counters adaptation by supplying a constantly updating tactile-proprioceptive stream: subtle variations in contact pressure, skin stretch and joint angles maintain afferent discharge rates without triggering habituation and preserve sensory precision over the full duration of the session.

Second, postural drift. Static balance involves continuous micro-corrections around a postural set-point, with center-of-pressure fluctuations occurring at 0.5–2 Hz during quiet standing (Winter, 1995). Without structured movement these corrections accumulate as unpredictable postural drift, increasing proprioceptive prediction-error variance and introducing noise into the very channels DPSA relies upon. The differentiable trajectory is proposed to convert those necessary balance adjustments into a predictable pattern, so that the movement may function as the correction, turning destabilizing drift into a structured sensory stream with low temporal entropy.

Third, epistemic value. From an active-inference perspective, movement serves both pragmatic and epistemic functions (Friston et al., 2012). Continuous motion generates informative sensory flow through variations in tactile pressure gradients, joint angles and weight distribution. That flow is proposed to confirm predictions about body-environment coupling and to maintain the epistemic engagement of the generative model. Static postures provide minimal epistemic value once initial contact is established and may increase uncertainty as adaptation progresses. Continuous differentiable motion is proposed to occupy a middle ground between static under-stimulation, in which the sensory stream adapts, and rapid or segmented movement, in which abrupt transitions generate large corrective updates. The claim concerns sensory renewal and the magnitude of prediction error, not energetic cost.

Coupling whole-body weight shift to hand movement amplifies these effects substantially and introduces a further stabilizing dimension. As the body’s center of mass moves in phase with the elliptical fingertip trajectory, tactile and proprioceptive cues evolve in parallel across multiple body segments: fingertips, wrists, shoulders, hips, knees and feet all contribute to a globally consistent sensory stream. The coupling generates a stable multisensory configuration in which both the spatial form and the temporal pacing of the movement remain consistent across the whole postural chain. Whole-body motion is proposed to provide an organized structure that minimizes competition among sensory channels, and so to reduce spontaneous attentional drift. Because the trajectory is generated by center-of-mass modulation rather than by isolated distal-limb effort, its sensory consequences remain globally predictable at the postural level, which further reduces the volatility of ascending prediction errors. Variability is proposed to fall not through effortful control but through the movement itself, which, distributed across the whole postural chain, may constrain fluctuations at several levels at once, creating a sustained low-variance sensory flow that deepens progressively with each cycle. Section 4.4 turns to the temporal organization this movement must have.

### 4.4 Temporal architecture and rhythmic entrainment

The temporal architecture of the movement provides a further stabilizing layer at the neural level. Stable rhythmic motion lowers computational demand by allowing internal timing models to govern movement without constant top-down correction. Once a predictable rhythm is established, the supplementary motor area, basal ganglia and cerebellar loops maintain timing with low variability (Swinnen and Wenderoth, 2004; Coull et al., 2011), and the need for prefrontal monitoring is predicted to decline. Beta-band synchrony is the electrophysiological signature of this procedural regime. Disruptions in smoothness produce transient increases in frontal theta activity, marking short-lived re-engagement of executive oversight (Engel and Fries, 2010), while sustained smooth movement maintains the beta-dominated state in which timing is automatic and cognitive cost is minimal.

Cycle periods of the order of tens of seconds, such as those observed in unpaced DPSA practice, fall within the infraslow cortical oscillation range associated with large-scale network transitions and autonomic coupling (He et al., 2008; Hiltunen et al., 2014). We note the overlap as context, not as evidence that any particular period is privileged. The infraslow band is where cerebellar error correction operates in continuous movement (Ivry and Spencer, 2004) and where respiratory-cardiac coupling stabilizes without forced synchronization (Yasuma and Hayano, 2004); it also lies below the theta band associated with episodic memory and executive monitoring. Whether the tempo that emerges reliably falls within that range, and whether coincidence with these rhythms confers any advantage, are empirical questions (Section 6), not design specifications. What the framework requires is only the property established above: that a predictable rhythm, whatever its period, allows timing to be maintained without counting, estimation or deliberate pacing, consistent with work on how low-frequency neural oscillations support dynamic attending in temporal context (Henry and Herrmann, 2014). Different levels of the cortical hierarchy operate at different characteristic timescales, and that is itself a fundamental organizing principle of neural architecture (Kiebel et al., 2008).

### 4.5 Computational framework and active inference

The neural and physiological mechanisms described above find their unifying explanation in a single computational framework: the free-energy principle and active inference. On this account the brain implements a hierarchical generative model that continuously infers the causes of its sensory input and reduces the mismatch between predicted and observed signals (Friston, 2010). Prediction error, the difference between what is expected and what arrives, is the currency of the system, and minimizing it is both the goal of perception and the driver of action. Active inference extends the account to action, proposing that organisms resolve proprioceptive prediction error directly, fulfilling motor intentions by moving in ways that generate the sensory consequences they expect instead of by issuing motor commands in the classical sense (Friston et al., 2011, 2016). DPSA is proposed to instantiate that principle: the differentiable trajectory is not commanded from above but is hypothesized to emerge from continuous resolution of proprioceptive prediction error at the sensorimotor boundary.

Sensory precision, expressed formally as π = 1/σ^2^ (the inverse variance of prediction errors), determines how strongly different channels influence the generative model. One contribution to the model’s objective can be represented by the precision-weighted prediction-error term L = ε^T^Πε, where ε denotes the prediction error vector and Π the precision matrix. That quantity is the size of incoming sensory mismatches, weighted by their estimated reliability. By dampening erratic visual and auditory input and replacing it with a continuously confirmed tactile stream, DPSA is predicted to reduce that quantity progressively with each cycle, allowing the generative model to settle without sustained corrective effort. Whether this corresponds to reduced executive correction is a separate empirical prediction, tested by the measures set out below; the formulation itself does not establish it. Under DPSA, precision is predicted to increase for tactile-proprioceptive channels while high-variance visual and auditory signals are down-weighted, and precision-weighted prediction error is predicted to decrease across cycles, supporting rapid convergence within each slow tactile-proprioceptive loop.

### 4.6 Laminar architecture and hemispheric coherence

At the laminar level, superficial pyramidal populations encode prediction errors while deep-layer pyramidal neurons encode conditional expectations, and reciprocal message passing between the two populations implements precision-weighted updating (Bastos et al., 2012; Parr and Friston, 2018). The upper layers report what went wrong, the lower layers hold what was expected, and the exchange between them determines what the brain does next. Attention on this account is the selective amplification of high-precision signals (Feldman and Friston, 2010), and DPSA is designed to engineer that amplification from the bottom up. Under DPSA the bilateral anchors are predicted to generate error signals of comparable amplitude and variance across hemispheres, mitigating lateralized bias. Callosal pathways promote interhemispheric coherence during bilateral sensorimotor processing, keeping early sensory predictions mutually consistent (Banich, 1998). Smooth proprioceptive inflow, stabilized by the slow-cycle loop, may further enhance the precision of internal body-state predictions within cerebellar and parietal circuits. Higher-order regions are therefore predicted to receive error signals that are already balanced and low in variance, allowing the hierarchy to settle into a stable equilibrium in which high-precision channels dominate and cross-modal competition is reduced, without recruiting the circuits that resolve conceptual and evaluative inference.

The slow continuous evolution of the fingertip trajectory is proposed to supply the kind of predictable input that may allow reduced firing variability and lower corrective load, and the absence of abrupt transitions is predicted to lower the frequency and magnitude of corrective updates. Continuous curvature is proposed to generate predictable novelty, delivering a sensory stream hypothesized to be varied enough to sustain epistemic engagement and prevent adaptation, yet predictable enough to maintain low prediction error and minimal corrective cost.

The resulting state is indexed by dynamical stability, that is, by reduced variability of the sensorimotor state trajectory over time, measured through cycle-duration coefficient of variation, largest Lyapunov exponent and center-of-pressure variance. These index the regularity of the movement itself. They are preferred to indices of neural signal diversity, which track global conscious level rather than attentional configuration. Section 4.7 considers the energetic consequences that would follow if the configuration were achieved, and the limits of what may be inferred from them.

### 4.7 Energetic consequences of the proposed network configuration

This section considers how the configuration proposed above would be expected to alter the distribution of neural recruitment across systems. The account is directional, not quantitative. The active-inference formulation proposes a relationship between precision-weighted prediction error and processing regime, but it is not a quantitative model, and reduced prediction error does not by itself entail reduced metabolic cost. Energetic organization is treated throughout as a predicted consequence of network recruitment.

Regional cerebral metabolism differs in mode as well as in magnitude, and the two are not tightly coupled. Aerobic glycolysis, glucose metabolized in excess of that predicted from oxygen consumption, is elevated in medial and lateral parietal and prefrontal cortex and falls below the brain mean in the cerebellum and medial temporal lobes. Sensory cortices combine high rates of glucose and oxygen consumption with low aerobic glycolysis, and aerobic glycolysis is not strictly related to total metabolic rate (Vaishnavi et al., 2010). Metabolic mode therefore cannot be read off total metabolic rate, and the prediction developed here concerns mode. Aerobic glycolysis appears instead to index biosynthetic and plasticity-related processes: during visual-motor adaptation it rises in task-relevant tissue, and the magnitude of that shift correlates with individual differences in adaptation rate (Shannon et al., 2016).

This yields a constrained and directional prediction. If the configuration proposed above alters the balance of recruitment across sensory, motor, cerebellar and association networks, the expected signature is a redistribution rather than a reduction: sustained engagement of task-relevant somatosensory and motor systems alongside reduced recruitment, relative to matched active controls, of prefrontal systems supporting deliberate monitoring and correction. A global reduction in metabolic activity would not support this account, and might instead indicate nonspecific relaxation, reduced arousal or disengagement. A second prediction cuts against any simple assumption that less activity is better. During early acquisition, while the bilateral relation is still being learned, aerobic glycolysis should increase in motor and cerebellar regions, declining as the coordination is internalized. Novice and experienced performance may therefore differ in direction, not merely in degree.

Two levels of test follow, of unequal accessibility. Portable functional near-infrared spectroscopy can examine whether dorsolateral prefrontal hemodynamic activity decreases while activity in accessible sensorimotor cortex is maintained. That dissociation is more informative than any general decrease, because it distinguishes altered allocation from simple hypoactivation. The method cannot reach the cerebellum or other deep structures, and it cannot quantify aerobic glycolysis, which requires positron emission tomography combining fluorodeoxyglucose and ^15^O measurement. The metabolic-mode hypothesis is accordingly a longer-term test, not a requirement for initial validation of the framework. Reduced total cerebral metabolism is not required, and its absence would not falsify DPSA; absence of the predicted prefrontal–sensorimotor dissociation under well-matched active controls would challenge this component of the mechanism.

### 4.8 Oscillatory and temporal coordination

The configuration described above is predicted to produce a characteristic and testable spectral signature at the oscillatory level.

Oscillatory rhythms are not incidental to the brain’s computations. They are how different levels of the hierarchy communicate at different speeds. Sustained beta-band activity specifies a stable predictive motor context (Engel and Fries, 2010), while gamma-band transients encode residual feedforward error signals (Bastos et al., 2015). This frequency-specific division aligns with the communication-through-coherence framework, in which stable beta-mediated feedback predictions are dynamically integrated with gamma-mediated error updating (Fries, 2005); this beta–gamma division of labor is a canonical organizational motif for hierarchical predictive control in cortical circuits (Bastos et al., 2012; Michalareas et al., 2016). As successive revolutions accumulate during the fixed-duration anchoring phase, cerebellar forward models are hypothesized to forecast the next tactile sample repeatedly, and the continuous curved motion is designed to deliver it reliably, progressively confirming those predictions with each cycle. The alignment between predicted and actual sensory outcomes should tighten progressively with each cycle. This is the proposed mechanism by which attentional stability deepens over time rather than merely arising, and does so without any increase in cognitive effort. DPSA is therefore proposed to exemplify embodied inference in a direct form: action continuously realizes the sensory consequences prescribed by the generative model, and stability follows from that realization.

The oscillatory signature of this configuration is measurable and distinctive, and the consequences of the configuration are not confined to sensorimotor circuits: they propagate upward into the interoceptive hierarchy, the autonomic system and eventually the body’s whole physiological state. Interoceptive signals are themselves understood within predictive-processing accounts as subject to precision-weighted inference rather than passive monitoring (Seth, 2013; Critchley and Garfinkel, 2017), which is the sense in which stabilization at the sensory boundary is proposed to propagate inward.

### 4.9 Autonomic and interoceptive propagation

Two propagation questions arise, one within a session and one across sessions. The within-session question is taken up below. The across-session question concerns a modulatory layer that sets the conditions under which practice takes place. Daily variations in cortical excitability, autonomic balance and tactile discrimination thresholds follow circadian rhythms that form part of the physiological context in which each DPSA session begins (Schmidt et al., 2007; Scheer et al., 2010). These rhythms are not proposed to alter the protocol’s core mechanisms, but are predicted to influence the baseline precision landscape from which each session starts. Within predictive-coding accounts the generative model maintains slow-timescale priors reflecting fluctuations in metabolic availability, autonomic tone and cortical gain, all of which follow circadian patterns. When DPSA is practiced at a consistent daily time, the internal milieu of arousal level, somatosensory clarity and autonomic tone is predicted to be more stable across days, potentially reducing state-dependent variability in the tactile-proprioceptive precision landscape and enabling quicker convergence toward the low-entropy attractor. Circadian alignment is therefore proposed as a modulatory state-condition and not as a core stabilizing mechanism. It would stabilize the physiological background against which bilateral anchoring and continuous movement operate, reinforcing shallow-hierarchy dominance and reducing the computational cost of maintaining coherent sensorimotor inference across sessions.

The stability established within fast sensorimotor circuits is not predicted to remain confined there. As prediction error is hypothesized to be resolved at the sensory boundary, fewer unresolved discrepancies are correspondingly predicted to propagate upward into higher-order control networks, reducing the demand for corrective intervention at slower timescales. In the interoceptive hierarchy, predictions in the mid-insula and anterior cingulate cortex (ACC) compete for precision with ascending sensory prediction errors. When DPSA reduces exteroceptive volatility, through predictable proprioceptive flow and stable center-of-mass dynamics, fewer ascending demands are hypothesized to fall on those higher-level regulatory circuits. The reduction is predicted to allow interoceptive predictions to assume greater precision, potentially increasing parasympathetic influence through vagal efferents and promoting autonomic quieting. Seen from within the active-inference framework, the embodied effects of DPSA are proposed to arise not from direct modulation of visceral signals but from minimizing competing exteroceptive volatility, which is in turn expected to reduce corrective demand on homeostatic regulation within the insula–ACC–vagal axis.

The settled state DPSA aims to produce is hypothesized to reflect reduced corrective demand rather than active inhibition. Stability at the sensory boundary should propagate upward through the hierarchy, reducing the exteroceptive noise that would otherwise demand corrective engagement at successively higher levels, and producing a progressive physiological stabilization that deepens with each cycle, without deliberate top-down regulation and without sustained metacognitive monitoring. Every step in this propagation account remains a theoretical prediction. None has been directly demonstrated. The chain requires empirical verification at each level, and setting it out explicitly is what defines the experimental program that will determine whether the proposed chain holds. Section 4.10 sets out how much confidence each layer of the account can bear.

### 4.10 Levels of claim and the inferential architecture

Before proceeding to the canonical protocol, it is worth distinguishing the claims made above by their proximity to empirical test. This transparency is specifically invited by the Hypothesis and Theory format. Four mechanistic claims sit closest to test. They are not the four defining constraints set out in Section 3, but proposals about how the paradigm is expected to operate within them: that bilateral simultaneous anchoring produces symmetric precision weighting; that sensory gating elevates somatosensory channel gain; that continuous differentiable motion prevents adaptation and structures postural correction; and that the temporal organization of the movement is compatible with infraslow oscillatory ranges. Each is directly testable through the control conditions specified in Section 5.2.

The derived claims, that these four mechanisms interact to confine prediction-error resolution to fast sensorimotor circuits and so produce a low-entropy attentional regime, follow theoretically if the mechanistic claims are confirmed, and are testable through the neuroimaging and electrophysiological predictions in Section 6. The speculative extensions, namely redistribution of metabolic mode, circadian modulation across sessions and autonomic propagation through the interoceptive hierarchy, are theoretically motivated and internally coherent, but rest on longer inferential chains and will require dedicated experimental programs to evaluate. All three levels of claim are presented as components of a unified theoretical framework, but they are not equally proximate to the experimental tests specified below. We set the architecture out at this level of detail because that is what a Hypothesis and Theory contribution is for: specifying the inferential chain before it has been empirically resolved, so that the predictions it generates can determine which links hold and which require revision.

## 5 The canonical DPSA protocol

### 5.1 Postural configuration, sensory anchors, and environmental conditions

The mechanistic account above specifies why each element of DPSA is proposed to contribute to attentional stability. This section describes how those elements are operationalized: the precise postural, sensory, kinematic and temporal parameters through which the framework becomes a reproducible experimental paradigm. Every specification here is motivated by the account that precedes it. The protocol is an operational expression of those mechanistic principles, not a collection of arbitrary procedural choices.

The participant stands barefoot on a stable, uniform surface with feet parallel and shoulder-width apart. Barefoot contact provides a consistent somatosensory reference at the ground interface, extending the proprioceptive field beyond the hands and contributing to the globally consistent sensory stream generated across the postural chain. The knees remain softly flexed, the spine elongated, and the head balanced naturally over the sacrum, maintaining postural neutrality without rigidity. The room is quiet apart from the ambient audio described below, with extraneous sound below 40 dB, a temperature between 18 °C and 25 °C, and visually neutral surroundings. Eyes are gently closed.

Auditory gating is achieved by masking rather than occlusion. Silence is not a neutral condition in a laboratory: an unexplained quiet room invites monitoring of the environment, which is the form of executive engagement the paradigm is designed to avoid. A continuous low-salience auditory background is therefore played in the room throughout the session. It reduces competing environmental and semantic input without removing audition, and provides a stable acoustic field in place of an unpredictable one. The recording consists of slowly evolving, continuously flowing ambient music without strong beats, metrical structure, melodic development or semantic content, so that it offers nothing to entrain to and nothing to attend to. It is played through room speakers. No earplugs or earbuds are used, since occlusion would work against the ambient bed and would introduce an additional tactile contact at the head. These conditions are methodological requirements, not comfort measures: they implement the paradigm’s sensory gating, elevating the relative gain of tactile and proprioceptive channels by removing visual input and reducing unpredictable auditory competition. Participants are positioned in individual spaces without social interaction, which minimizes unstructured social and environmental variability across sessions.

The bilateral sensory anchors are formed by lightly joining the index and middle fingertips of each hand. The contact is light; its purpose is not to apply pressure but to make each pair a single perceptual unit. Attention rests on the external surfaces of these units as combined multi-dimensional reference points: their position in space, the direction and quality of their movement, tactile contact with air currents and micro-vibrations; pressure gradients; temperature; and subtle shifts in proprioceptive tone. Only the index and middle fingertips make contact. The remaining fingers stay relaxed and outside the attentional field. The configuration is proposed to establish two stable, high-density somatosensory reference points, one per hemisphere, engaging the bilateral receptive fields of primary somatosensory cortex. Attention is distributed evenly between the left and right anchors, without emphasis on internal differences within each pair. The two anchors are engaged simultaneously and symmetrically, and the relation between them, rather than either alone, is the proposed object of attention (Section 4.1). Anchoring at the fingertips requires no fine manipulative skill and no equipment.

The demands of the paradigm lie not in the anchors but in the movement that carries them: whole-body weight transfer, knee flexion and extension, torso rotation and substantial lateral excursion of the hands, all sustained while standing with the eyes closed. Any claim of translational reach is therefore governed by the eligibility criteria set out below, and not by the simplicity of the anchor configuration. Figure 1 illustrates the bilateral anchor configuration and its relationship to the elliptical fingertip trajectory.

**FIGURE 1:**
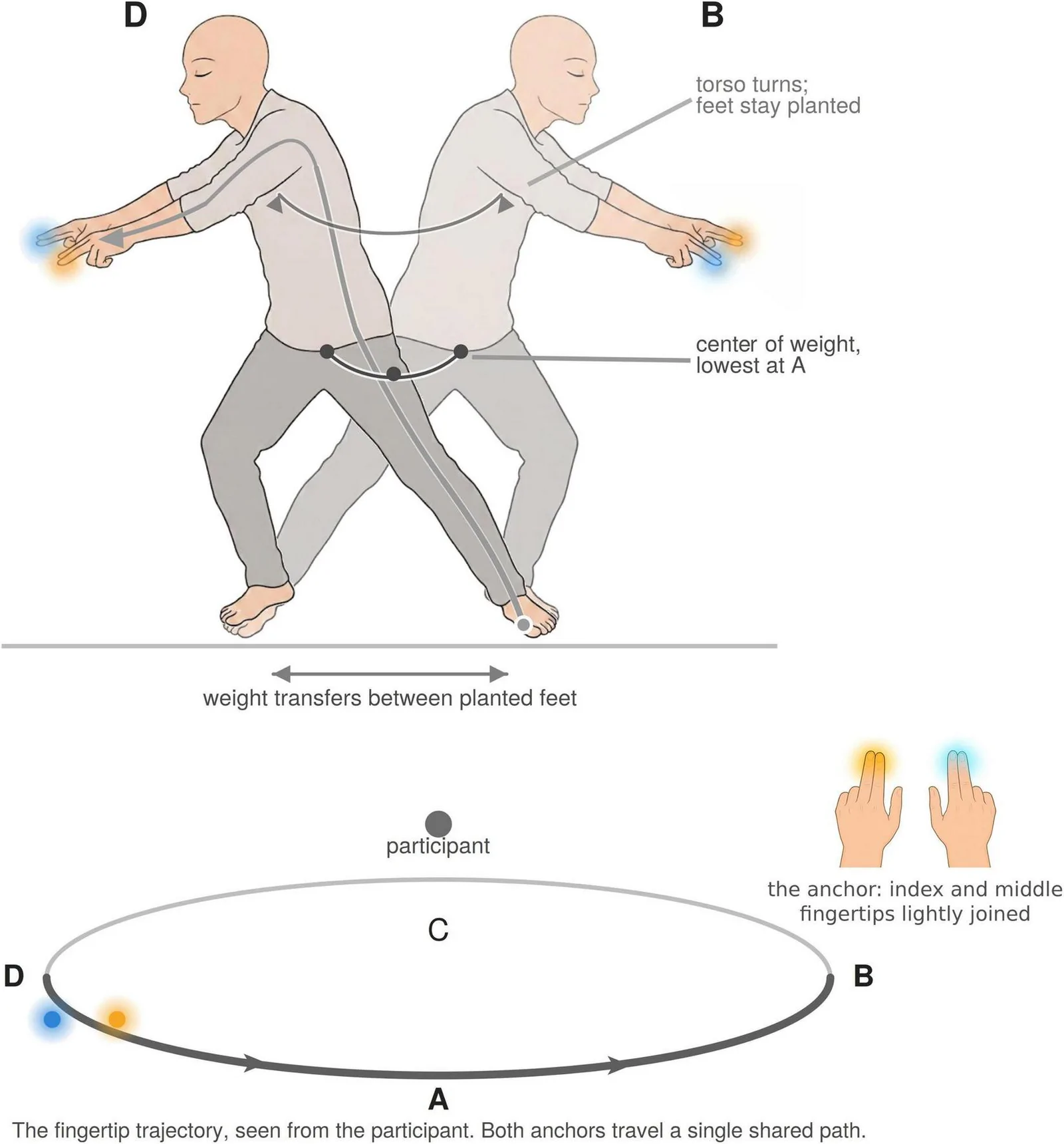
Dual-point sensory anchoring: whole-body movement and the fingertip trajectory. **Upper:** a lateral view of the standing posture at two positions on the path. The solid figure shows position D, at which the torso is turned to one side and the weight is carried into the weight-bearing leg with the contralateral leg extended; the ghosted figure shows position B, the corresponding extreme on the other side. The transition between the two involves torso rotation, lateral translation and redistribution of weight while both feet remain planted in the same positions, as indicated by the upper arc drawn at chest height. The gray line traces the proximal-to-distal kinetic chain by which the movement is generated, originating at the supporting foot and propagating through the leg, hip, trunk, shoulder and arm to the fingertips; it is drawn on the solid figure only, and the trunk is depicted as a transmitting link within this chain rather than as an independent initiator of the movement. The marked path at pelvis height shows the center of weight between the two positions, running near level but reaching its lowest point at A. The line at the base indicates the transfer of weight between the two planted feet. **Lower:** the fingertip trajectory, drawn from the participant’s own vantage, with the body positioned at the near edge of the ellipse. Both anchors travel a single shared path rather than separate trajectories, and are shown here at position D. The bold arc marks the half of the circuit traveled from D through A to B, with the return through C shown faint. Position A is the far point of the path, reached with the torso square to the front; C is the near point, at which the hands are drawn in close to the body at approximately heart level and the torso is again square; B and D are the lateral extremes, at which the torso is turned to the corresponding side. Travel proceeds continuously in one direction throughout a session, and the direction is held constant within participants. The lateral extent of the path is substantially greater than its near-to-far extent, and the ellipse is drawn to reflect this. The ellipse is schematic: no fixed trajectory dimensions are prescribed. Amplitude is calibrated individually to each participant during familiarization, constrained by the requirement that the movement remain continuous and within stable postural limits, and the resulting amplitude and cycle duration are recorded for each participant rather than set in advance. The detail at upper right shows the anchor itself: the index and middle fingertips of each hand lightly joined, with the amber and blue fields depicting the participant’s sustained allocation of tactile attention to each fingertip pair. These fields do not correspond to external stimuli. Together the two views illustrate the core structure of DPSA: bilateral sensory anchors at the fingertips, the whole-body movement that carries them, and the uninterrupted continuity of the path they trace. Figure prepared with the assistance of Anthropic Claude.

This protocol is a standing one. Continuous transfer of the center of mass through the lower limb is not incidental to the paradigm; it generates the whole-body proprioceptive stream within which the fingertip anchors are embedded. No seated or otherwise modified form of the protocol has been developed or evaluated. We therefore make no claims regarding such variants, and note that whether the paradigm can be realized in a configuration that does not load the lower limb is an open question, not a matter of adaptation.

Participants are adults able to stand unsupported for 30 min without discomfort, that is, for twice the duration of a session. Screening excludes a history of falls within the preceding 12 months, diagnosed vestibular disorder, orthostatic intolerance, and any condition materially affecting standing balance. Where studies include older adults, standing balance is assessed at baseline using a standard clinical measure and reported with the participant characteristics. These requirements follow the conventions established in research on standing whole-body movement practices, which have been delivered under supervision to older and clinically compromised populations in randomized trials (Yeh et al., 2004). DPSA differs from those protocols in one respect material to safety: deliberate eye closure removes visual input, while auditory masking stabilizes the acoustic environment without removing audition. The principal established postural concern is therefore eye closure, which measurably reduces standing stability. The screening criteria are set accordingly, and are more conservative than those typical of the movement-practice literature.

Sessions are conducted in the presence of an observer whose role is confined to safety. The observer remains seated, silent and stationary throughout, positioned outside the visual field in conditions where the eyes are open, and does not speak, move or take notes during the anchoring phase. No feedback concerning performance is given at any point. Before the first session participants are told that the observer is present only in case of loss of balance, that no aspect of their performance is being assessed, and that performance is not judged for elegance, depth or speed; they are asked to follow the protocol itself as described. This is stated explicitly because the intended effect of the paradigm concerns the absence of self-monitoring, and the belief that one is being evaluated is among the more reliable ways to introduce self-monitoring. Participants are reminded before each session that they may open their eyes, reach for a stable support positioned within reach, or discontinue at any point and without explanation. That support is not used unless required. Any report of dizziness, unsteadiness, visual disturbance or discomfort ends the session. Whether the observer’s presence nonetheless introduces self-monitoring is measured rather than assumed: a perceived self-consciousness item is included alongside the perceived-effort and engagement ratings.

### 5.2 Movement, temporal architecture, and control conditions

Each fingertip anchor traces a slow, continuous, closed-loop elliptical path, a Differentiable Fingertip Trajectory, in which position, velocity and direction change smoothly and without abrupt transitions at any point. The trajectory is generated not by isolated arm movement but by distributed adjustments across the whole postural chain, each individually small: feet, knees, hips, spine, shoulder girdle, elbow and wrist all contribute. Both hands travel a closed path in a horizontal plane, extending from close in to the body at approximately the level of the heart at its nearest point, to full arm extension at its furthest, and to either side of the midline at its lateral extremes. That path lies in front of and beside the body; it does not encircle the body.

Two features of the resulting coupling bear directly on the whole-body claim. First, the hands hold a near-constant absolute height throughout the circuit, tracing a level plane rather than rising and falling. The apparent change in the height at which the hands sit relative to the torso arises because the body rises and falls beneath a stationary hand-plane, through knee flexion and extension. The center of weight is therefore lowest where the hands are furthest from the body and highest where they are drawn in closest, completing one such cycle per circuit. The level at which the hands sit against the torso consequently varies inversely with the center of weight, sitting higher against the torso when the body is at its lowest and lower when it is at its tallest. In an experienced practitioner this excursion may span from approximately the sternum at the sunk point of the cycle to the upper abdomen at the tallest; a smaller range is expected initially, and the amplitude is calibrated individually rather than prescribed. It is recorded for each participant, but it is not a performance measure, and a greater excursion is not treated as better practice. The vertical excursion exists to generate the continuous proprioceptive stream within which the anchors are embedded. What the paradigm concerns is the quality of that coupling, not the depth achieved.

Second, the feet remain planted in fixed positions throughout the circuit. Within this stable base the torso turns and translates laterally as weight transfers continuously between the two legs, carrying the hands toward either side of the body through integrated whole-body movement instead of independent arm reaching. The torso is square to the front at the near and far points of the path and turned toward the corresponding side at the two lateral extremes.

The circuit is entered and left from a defined posture. At the start of the calibration phase the practitioner adopts a preparatory posture: arms extended forward, torso square to the front, legs close to straight and the center of weight high. The preparatory posture shares the horizontal position of station A but not its height, and is not itself a station on the path. From it the center of weight is lowered slowly through knee flexion until the body reaches station A, the far point of the circuit at which the center of weight is low; the first turn toward one side begins only once station A has been reached. The circuit is left by the reverse sequence. The final revolution returns to station A at the lowered center of weight, the movement slows to a stop, and the legs straighten so that the center of weight rises back into the preparatory posture, concluding with the fade at 14:50–15:00. Entry and exit are therefore vertical movements between the preparatory posture and station A, and the horizontal path begins and ends at that station.

Three qualifications govern how this specification should be read. First, the four positions are descriptive landmarks, not discrete targets: no pause occurs at any of them, and the path is executed as smoothly as the practitioner can achieve. They are identified here, and in Figure 1, only because a continuous path cannot otherwise be described in text. Second, and more importantly, arrival at these positions is not the object of the practice. The object is what occurs between them: the continuous multi-dimensional sensory input at the two anchors, and the evolving relation between them, attended to at every moment of the circuit. The path exists to generate that stream, and a practitioner who executes the geometry accurately while attending elsewhere is not performing the paradigm.

Third, the tempo is not externally prescribed or consciously counted; it arises from how the participant engages with the movement. Two candidate determinants are identified for measurement rather than assumed: the time required to register and integrate the sensory input described above, and the delay of transmission along the kinetic chain from foot through leg, hip, trunk and arm to the fingertip. This simultaneous whole-body coupling is treated as essential, not incidental. It is proposed to be the mechanism by which the globally consistent multisensory stream described above is generated, engaging proprioceptive evidence from several body segments at once instead of from the hands alone. The perceptual reference throughout is the evolving fingertip-centered sensorimotor stream: what the anchors are doing and sensing in space, not the underlying postural mechanics that generate the motion.

The trajectory is performed at the tempo the practice itself requires, and no cycle duration is prescribed. The object of the practice is the evolving sensory content at the anchors, not arrival at any point on the path. The pace is therefore set by the time that attending takes: registering the changing tactile and proprioceptive relation, and allowing it to inform the movement that follows. Transmission along the kinetic chain from foot to fingertip imposes a lower bound on how rapidly the coupling can be maintained, but the resulting pace is proposed to be set primarily by attentional demand rather than by biomechanics alone. The tempo is therefore expected to differ between individuals and to change with practice, and it is not monitored or counted during the session. The slow cycle is an emergent consequence of this arrangement, not a prescribed tempo. The shift of the body’s center of mass carries the hands through the path without conscious pacing, counting, or deliberate effort to maintain a rhythm.

No particular duration is proposed as correct or optimal, and participants are not expected to converge on a fixed personal tempo. Pace may vary across and within participants as the evolving sensory content, coordination demands and postural conditions change. Cycle duration, its variability, corrective events and completed revolutions are recorded as empirical characteristics and not treated as performance criteria. Moving a large distributed mass smoothly under balance constraints is one candidate influence on the pace that results: on this account the slow cycle is what smooth whole-body motion at this scale would be expected to produce.

During preparatory familiarization sessions, participants practice synchronizing fingertip motion with whole-body weight transfer until a stable coordination pattern is established; once internalized, the movement proceeds without deliberate tracking of its speed. Familiarization conveys the coordination pattern only. No numerical target, counted rhythm, auditory pacing signal, metronome or externally demonstrated cycle duration is provided at any point, so that the tempo a participant settles into is not taught. This matters for Prediction 10: were any pacing information supplied during familiarization, the pace a participant settles into could reflect instruction rather than emergence. The main anchoring phase comprises however many revolutions the emergent pace produces within its fixed duration. The number completed is recorded rather than prescribed, and provides repetition for cerebellar forward models to progressively refine their predictions and, on the account developed above, for attentional stability to deepen across cycles.

Breathing proceeds naturally throughout and is explicitly not synchronized with the movement cycle. No attempt is made to regulate respiratory rhythm, depth or phase. This preserves the independence of the slow-cycle loop from faster physiological processes and avoids introducing an additional cognitive instruction into the protocol. Leaving respiration unregulated is itself a design choice: it distinguishes DPSA from breath-focused practices and is intended to ensure that any stabilizing effects can be attributed to the movement architecture and not to deliberate physiological regulation.

The session follows a fixed temporal structure of 15 min, organized into four phases with smooth audio-cued transitions (Table 2). The opening calibration phase of approximately 3 min establishes the postural and attentional configuration: the participant finds their stance, forms the bilateral anchors, and begins the elliptical movement at a comfortable pace. The transition-in phase of approximately 2 min allows the movement to settle into its natural tempo and the attentional field to stabilize around the two anchors. Together these opening 5 min bring the practitioner into the anchoring phase gradually rather than abruptly, so that the whole body is already moving in a settled coordination pattern before the main phase begins. The reasoning parallels that governing the fades: an abrupt onset would create a discrete event of the kind the protocol is designed throughout to avoid. The main anchoring phase of approximately 8 min is the core of the session, with continuous bilateral movement and no additional instruction; the participant attends to the fingertip anchors as attentional stability is proposed to deepen progressively across cycles. The transition-out phase of approximately 2 min allows sensory activity to settle before the session closes.

**TABLE 2:** Timing structure of the canonical DPSA protocol.

Phase	Duration (mm:ss)	Fade interval (mm:ss)	Amplitude range	Function
Intro	0:00–0:10	0:00–0:10	0% → 100%	Establish baseline tone salience
1 Calibration	0:10–3:00	2:50–3:10	100% → 10% → 100%	Cue transition into Phase 2
2 Transition-In	3:10–5:00	4:50–5:10	100% → 10% → 100%	Cue transition into Phase 3
3 Anchoring	5:10–13:00	12:50–13:10	100% → 10% → 100%	Cue transition into Phase 4
4 Transition-Out	13:10–15:00	14:50–15:00	100% → 0%	Final fade and session completion

Total duration: 15 min. Amplitude envelope: linear or gentle sinusoidal. Sound type: continuous ambient music, without strong beats, metrical structure or semantic content. Amplitude changes mark phase boundaries; the recording itself carries no internal rhythmic structure that could pace the movement. Fade intervals straddle the phase boundary rather than belonging to either adjacent phase, and are included within the fixed 15-min duration. A fade of approximately 20 s therefore defines the window within which each phase transition may be made, at any point the practitioner chooses; the opening and closing fades span 10 s. The phase durations given are nominal, and no transition is required at a specific instant.

Phase transitions are signaled within the same continuous recording by a gradual reduction and restoration of amplitude, and not by any discrete cue. The reasoning parallels the continuous-curvature constraint on the movement: a discrete signal creates a discrete event, and with it a moment of orientation and a reason to hurry, whereas a fade carries the same information without an abrupt transition. Each fade between phases spans approximately 20 s, and the practitioner may make the transition at any point within that window; there is no moment at which the change is required. The opening and closing fades, which begin and end the session rather than marking a transition between phases, span 10 s. The baseline playback level is set once during familiarization, to a level the participant reports as comfortable, and is held constant, outside the predefined fade intervals, for that participant across all sessions and conditions, so that the auditory environment is identical throughout and cannot differ between conditions. The level used is recorded for each participant.

The design comprises DPSA together with six control conditions, seven conditions in total, and the controls fall into two families serving different purposes. The first family dissociates the defining constraints. The unilateral condition uses one hand only, matched in all other respects, and tests bilateral symmetry. The non-differentiable condition replaces the smooth elliptical path with an angular or segmented trajectory, and tests sensorimotor continuity. The eyes-open condition restores a standard visual environment, and tests sensory gating. The arms-only condition holds the trunk stationary and restricts movement to the arms, and tests the whole-body component of the first constraint. The movement-matched condition retains the movement while removing any instruction concerning the fingertips, and tests the contribution of anchoring itself. The second family addresses alternative explanations: a resting condition, standing with eyes closed and without movement, isolates what eye closure and reduced exteroceptive load produce on their own, and the movement-matched condition serves this family also, isolating what continuous bilateral movement produces in the absence of anchoring. Effects that survive both comparisons cannot be attributed to relaxation, eye closure, or rhythmic movement as such. Standing rest with the eyes closed is not a posturally neutral comparison, since visual deprivation increases postural sway; the condition therefore differs from DPSA in balance demand as well as in movement and attentional instruction. A seated resting condition would introduce a still larger postural difference from DPSA, so eyes-closed standing rest is retained, with its greater balance demand treated as a design limitation.

Expectancy is measured, not assumed. Because DPSA is a novel paradigm presented to participants as a candidate method for stabilizing attention, differential expectancy between the experimental and control conditions is a live alternative explanation for any effect observed, and one that a within-participants design does not remove. The Credibility/Expectancy Questionnaire (Devilly and Borkovec, 2000) is administered after the rationale for each condition has been given and before that condition is performed. Its two factors are scored separately and, as the original authors emphasize, not according to the apparent grouping of the items on the form: credibility is derived from the first three items, and expectancy from the fourth together with the two affective items. Expectancy ratings are entered as covariates in the primary analyses. The prediction is that the pattern of outcomes across conditions will not be reproduced by the pattern of expectancy ratings. If condition differences in the neural, autonomic and kinematic measures track expectancy ratings, the mechanistic account is not supported, whatever the direction of the effects.

Each condition generates a directional prediction capable of falsifying the corresponding mechanistic claim, and the pattern of selective degradation across the first family constitutes the systematic dissociation described in Prediction 9. The primary single-session test comprises four conditions — DPSA, unilateral anchoring, non-differentiable movement and eyes-open DPSA — and is implementable in one sitting. It is an initial test, not a complete dissociation of the four constraints, since it excludes the anchoring and whole-body comparisons; the remaining conditions are distributed across counterbalanced sessions. The design uses standard electrophysiological methods and portable neuroimaging compatible with standing movement. These include independent component analysis of continuous EEG data, which, combined with simultaneous electromyographic and kinematic recording, is used to identify and attenuate likely artifactual components (Makeig et al., 2004).

Attentional state cannot be measured directly during the anchoring phase without altering the state under investigation. The paradigm’s defining constraints remove the response channels an in-session probe requires. Both hands are anchored, so a manual response breaks the anchor. A verbal response recruits the conceptual processing that the framework proposes is disengaged. Any probe capable of prompting a report is itself exteroceptive input of the kind the sensory-gating constraint removes. A probe would also interrupt precisely the cumulative process that Prediction 5 concerns, so the measure would degrade the effect it was introduced to detect.

All in-session measurement is therefore passive, and three measures stand in place of direct report. A structured retrospective report is administered within 2 min of session end; in it, participants estimate the proportion of the session spent attending to the fingertip anchors, to the body and movement more generally, and to task-unrelated thought, and rate perceived effort, attentional engagement, perceived self-consciousness and credibility. This is retrospective and is treated as such: it is subject to reconstruction and to demand characteristics, and it is a secondary outcome. Electro-oculographic channels provide a continuous passive index of arousal, since slow rolling eye movements under closed lids mark the onset of drowsiness; their presence distinguishes the settled state the framework proposes from reduced vigilance, which is among the alternative explanations the design must exclude. Where a direct in-session attentional report is required, intermittent experience sampling is administered in a separate, dedicated session, on the explicit understanding that probed and unprobed sessions measure different states and that physiological outcomes from probed sessions are not interchangeable with those from the primary protocol.

The probed session is specified as follows. Four probes are delivered during the anchoring phase of that session at pseudo-random intervals, constrained so that no two fall within 90 s of one another and none falls within the first or last minute of the phase. Each probe is a brief rise in the amplitude of the ambient background already present in the room, distinguishable from the phase-transition fades by its duration rather than by any added tone, so that no new auditory element is introduced. On hearing it the participant states aloud a single word describing where attention was immediately beforehand, choosing among four responses: anchors, for the fingertips or the relation between them; body, for posture, weight transfer or the movement generally; elsewhere, for task-unrelated thought; and unsure. The response set is taught during familiarization and rehearsed until the choice is automatic, so that probing costs a word rather than deliberation. The primary measure is the proportion of on-anchor responses; the proportion of elsewhere responses provides the mind-wandering comparison. Probes are delivered identically in the DPSA and movement-matched conditions, so that the interruption is common to both and does not confound the contrast between them. Because a spoken response recruits the conceptual processing that the framework proposes is disengaged, this session is treated as a perturbed variant throughout, and its physiological measures are reported separately from those of the canonical protocol.

A final methodological note concerns measurement apparatus. DPSA requires continuous whole-body movement throughout the anchoring phase, and the protocol’s stabilizing effects are predicated on the participant experiencing minimal cognitive or physical constraint during the session. Wired EEG systems, motion capture systems requiring cable management, and electrode arrays that restrict postural freedom all introduce sources of attentional distraction and movement constraint that are theoretically incompatible with the paradigm’s design principles. Wireless and mobile measurement systems, including high-density wireless EEG, inertial measurement unit (IMU) arrays for motion capture and wearable HRV monitors, are therefore the preferred apparatus for empirical testing of DPSA.

The full recording montage is accordingly passive and is fitted before the session begins: wireless EEG with electro-oculographic channels; wireless surface electromyography at the neck, shoulder and forearm; a respiration belt; an electrocardiographic chest strap; inertial measurement units at the wrists, trunk and ankles; and either instrumented insoles or a force platform for center-of-pressure recording. The electromyographic and inertial channels are not incidental. Movement and muscle artifacts in ambulatory recordings can exceed cortical signals by one to two orders of magnitude, so independent component analysis with artifact classification is applied before any spectral or connectivity analysis, and simultaneous electromyographic and movement records are required to identify the components to be removed (Makeig et al., 2004). Respiration is recorded because high-frequency heart rate variability is strongly influenced by respiratory rate and depth; high-frequency heart rate variability is therefore reported alongside the root mean square of successive differences (RMSSD) rather than in place of it. Connectivity analyses are conducted in source space with a predefined artifact-rejection pipeline specified before data collection. Beyond preserving the integrity of the sensorimotor conditions, wireless systems also support ecological validity: they allow the paradigm to be studied in naturalistic settings beyond the laboratory, which bears directly on the translational program described in the Discussion. Where wired systems are the only option available, researchers should document any participant-reported awareness of equipment constraint and treat it as a potential moderating variable in the analysis.

## 6 Empirical predictions and testability

A theoretical framework stands or falls on the precision and falsifiability of the predictions it generates. DPSA generates specific, directional predictions about the neural, autonomic, hemodynamic and behavioral consequences of its four defining constraints. They follow from the mechanistic account developed above, and the primary predictions among them are testable using standard electrophysiological, portable neuroimaging and physiological methods. This section sets them out, identifies the control conditions through which each can be falsified, and considers the boundary conditions and translational implications of the framework.

Before the predictions are set out, four terms used throughout Section 4 require operational definition, since none is directly observable and each is indexed by measures rather than identical to them. Attentional stability denotes sustained allocation of attention to the fingertip anchors without intervening episodes of task-unrelated thought. Because the paradigm precludes direct in-session report (Section 5.2), it is indexed convergently rather than measured: by the variability of kinematic and postural measures, by the ocular and autonomic markers that distinguish sustained engagement from drowsiness, and by structured retrospective report. No single one of these is a measure of attention, and the framework does not treat any of them as one. Low-entropy state is a theoretical description of a progressively constrained distribution of sensorimotor states. In the initial studies it is provisionally indexed by cycle-duration coefficient of variation, largest Lyapunov exponent and center-of-pressure variance; these assess variability and dynamical stability rather than information-theoretic entropy directly, and the term is used in its theoretical sense throughout. Hierarchical confinement denotes the resolution of prediction error predominantly within somatosensory and cerebellar circuits rather than at prefrontal levels; it is indexed by the relative distribution of hemodynamic activity between dorsolateral prefrontal and somatosensory territory, not by any single regional value. Progressive deepening denotes directional change in these indices across the course of a session rather than a step-change at onset; it is indexed by the slope of each measure across the anchoring phase.

The distinction between indexing and identity matters here. Beta and gamma coherence, heart rate variability, hemodynamic signal and movement regularity are not measures of attention, and the framework does not treat them as such. They are proposed consequences of an attentional configuration whose direct behavioral signature cannot be obtained during the canonical protocol without perturbing it. Within the canonical unprobed protocol, attention is indexed convergently by the passive physiological and kinematic measures together with the structured retrospective report. Direct experience-sampling data are obtained only in the separate probed session, and are interpreted as evidence about attentional phenomenology under a deliberately modified condition, not as an outcome measure for the unprobed protocol. Where a prediction concerns attention itself, the probe data constitute the primary outcome and the physiological measures are convergent evidence; where a prediction concerns a physiological consequence, the relevant physiological measure is primary. Each prediction below names its primary outcome measure, the expected direction of change, its time course where one is specified, and the result that would falsify it.

Prediction 1 — Beta-band coherence. If the bilateral anchors are bound into a single relational structure across hemispheres, engaging homologous somatosensory territories through callosal pathways and sustaining a balanced bilateral precision landscape, then wireless EEG recordings during DPSA should show significantly strengthened beta-band coherence between homologous S1 and S2 regions compared to the unilateral, non-differentiable and eyes-open conditions. The enhancement should emerge within the first several cycles of the main movement phase and strengthen progressively as the session continues, reflecting the progressive deepening of attentional stability proposed above. Disruption of bilateral symmetry in the unilateral control should produce a measurable lateralization of coherence, with the hemisphere contralateral to the moving hand showing elevated activity relative to the ipsilateral hemisphere. The prediction is directional and specific, and it is distinguishable from what generic relaxation or bilateral stimulation paradigms would predict.

Voluntary movement produces event-related desynchronization in the beta band over sensorimotor cortex, so regional beta power and interregional beta coupling must be distinguished: desynchronization is a reduction in power within a region, whereas the prediction here concerns phase coupling between homologous regions across hemispheres. Interhemispheric coupling at approximately 20 Hz between left and right motor areas has been demonstrated during voluntary finger movement using measures insensitive to volume conduction (Nolte et al., 2004). The distinction is treated as a methodological constraint rather than a resolved question. Beta power and interhemispheric coupling are reported separately, so that a change in coupling cannot be attributed to a change in power. The primary connectivity measure is the imaginary part of coherency, which is insensitive both to zero-lag volume-conduction artifacts and to amplitude; it will also discard any genuine zero-lag coupling, and magnitude-squared coherence is reported alongside it for that reason. If the predicted increase in coupling appears only in amplitude-sensitive measures and not in the imaginary component, it should be treated as ambiguous rather than as evidence of interhemispheric integration, because power differences, volume conduction and genuine zero-lag coupling cannot then be cleanly distinguished. The prediction is falsified if interhemispheric beta coherence during DPSA does not exceed that observed in the comparison conditions, or if coherence fails to strengthen across the session: either result would indicate that bilateral anchoring does not produce the cross-hemispheric binding the framework proposes.

Prediction 2 — Prefrontal hemodynamic load. If DPSA confines prediction-error resolution predominantly to somatosensory and cerebellar circuits, then hemodynamic measures of dorsolateral prefrontal activity should show a reduction relative to baseline and relative to the eyes-open control condition. Portable fNIRS measures should show reduced oxygenation in dorsolateral prefrontal cortex during the main anchoring phase, while somatosensory signal remains stable or increases modestly. Conventional fMRI is incompatible with the standing protocol, since the participant must remain in continuous whole-body motion. Portable fNIRS permits measurement in an ecologically valid context, but its sensitivity is not equivalent to that of fMRI: penetration is limited to approximately 1.5–2 cm with spatial resolution of the order of 2–3 cm (Scholkmann et al., 2014), and anterior cingulate and cerebellar activity are therefore not measurable by this method. The prediction is accordingly restricted to accessible dorsolateral prefrontal and somatosensory territory, and the deeper components of the hierarchical-confinement account remain untested by it. Reduced higher-order activity with maintained or elevated sensorimotor activity would constitute the neural signature of hierarchical confinement, and would be directly distinguishable from the pattern produced by passive rest, in which both prefrontal and sensorimotor activity decline together. The prediction is falsified if prefrontal and somatosensory signals decline together, which would indicate nonspecific disengagement rather than redistribution, or if neither changes relative to the eyes-open condition.

Prediction 3 — Somatosensory gamma coherence. Under the sensory-gated configuration, gamma-band coherence within somatosensory cortex should increase during DPSA relative to the eyes-open control. The eyes-open condition reintroduces visual competition, which should reduce somatosensory gamma coherence and increase cross-modal signal variance. The magnitude of that difference, gated against ungated, provides a direct measure of the precision engineering that eye closure and controlled auditory masking accomplish. Scalp gamma during sustained whole-body movement is particularly susceptible to muscular contamination, so this prediction is technically more demanding than the beta-connectivity, kinematic and autonomic predictions, and is not among those implementable with standard equipment alone. If the difference is absent or reversed, the sensory-gating claim is falsified.

Prediction 4 — Autonomic regulation. Section 4.9 describes a mechanism by which stability established within fast sensorimotor circuits propagates upward into the interoceptive hierarchy: reduced exteroceptive volatility allows interoceptive predictions to assume greater precision, increasing parasympathetic influence through vagal efferents. If that mechanism operates, heart rate variability should increase during the main anchoring phase, relative both to baseline and to the non-differentiable trajectory control. High-frequency HRV (HF-HRV), the component most directly reflecting vagal tone, should show the largest increase, and that increase should correlate with the degree of beta coherence enhancement observed in EEG recordings. A correlation between the neural and autonomic signatures would provide convergent evidence for the hierarchical propagation mechanism and substantially strengthen the framework’s empirical standing. Because respiration is deliberately unregulated, respiratory rate and depth are modeled as covariates and the condition effect is required to survive respiratory adjustment. RMSSD is reported alongside HF-HRV, and neither is treated on its own as evidence of vagal propagation. The prediction is falsified if HF-HRV does not increase relative to the non-differentiable control. The propagation account in particular is not supported if autonomic change occurs without any relationship to the neural measures, a pattern that a general relaxation account would predict and that this framework would not.

Prediction 5 — Temporal dynamics of stabilization. The mechanistic account above proposes that attentional stability arises and deepens progressively with each cycle of the movement rather than appearing as an immediate step-change at session onset. This predicts a specific temporal profile in both neural and behavioral measures. The measures should change directionally across the fixed-duration anchoring phase. Analyses model elapsed time and normalized phase position, with cycle-based analyses reported secondarily, so that participants completing different numbers of revolutions remain comparable. The direction differs by measure. Interhemispheric beta coherence and HF-HRV are predicted to increase; cycle-duration coefficient of variation, center-of-pressure variance and the largest Lyapunov exponent are predicted to decrease, since greater stability corresponds to a narrower and less variable state trajectory.

The primary behavioral measure is the cycle-duration coefficient of variation derived from wrist-worn inertial sensors, with center-of-pressure variance from a force platform as a secondary measure where the recording environment permits. Cycle-duration CV is chosen on design grounds rather than on assumed effect size: it is computed within participants, requires no normative comparison, adds no instrumentation, and is defined identically across all conditions. Its sensitivity in this paradigm is unknown, so the first study should treat its variance structure as an estimand and report the observed within-participant distribution for later studies to be powered on. If the preregistered measures show no directional temporal development relative to the matched controls, the progressive-deepening claim is not supported. The form of any observed trajectory, whether linear, saturating or variable, is estimated rather than assumed in advance.

Prediction 6 — Specificity of local trajectory continuity. The non-differentiable trajectory control tests whether an unbroken path is specifically necessary or whether any bilateral movement produces comparable effects. The mechanistic account predicts that discrete transitions in the trajectory will produce detectable transient increases in frontal theta activity — the electrophysiological signature of executive re-engagement — that are absent during smooth continuous motion. Cerebellar forward model accuracy, indexed by the temporal precision of anticipatory motor adjustments, should also be lower in the non-differentiable condition. Equivalence between smooth and segmented trajectories would not support the proposed contribution of smooth local transitions. The condition bears on segmentation only: geometric closure, constant direction and the absence of inter-cycle reset require dedicated pause, reversal and open-path manipulations.

Prediction 7 — Post-session persistence. If DPSA produces a genuine shift in the precision landscape rather than a transient state dependent on the physical presence of the movement, then some degree of attentional and autonomic stability should persist for a measurable period after the session ends. Beta coherence and HRV measures taken immediately post-session and at 5 and 15 min post-session should show a gradual return toward baseline rather than an immediate collapse. The rate of return toward baseline provides information about the depth of the inferential shift that the session produces and may prove relevant to understanding the optimal frequency and duration of DPSA practice in translational contexts. The prediction is falsified if the measures return to baseline immediately at the cessation of movement, which would indicate a transient state dependent on the movement itself rather than a shift in the precision landscape.

Prediction 8 — Dose-response across sessions. If repeated engagement with the predictable curved motion strengthens cortico-cerebellar internal models through activity-dependent plasticity, then kinematic regularity, beta coherence and HRV should improve across sessions, following a learning curve consistent with motor skill acquisition. Naive participants should show high variability in their first sessions and progressively lower variability as the movement is internalized. Experienced practitioners should show faster convergence to the stabilized regime from session onset. This prediction is testable in a simple longitudinal design and distinguishes DPSA from paradigms whose effects do not accumulate with practice. The prediction is falsified if within-participant variability does not decline across sessions, or if naive and experienced participants show equivalent profiles from the outset: either result would indicate that the effects do not depend on model acquisition and would be better explained as immediate state changes.

Prediction 9 — Boundary conditions and selective degradation. DPSA makes claims about a specific combination of constraints. Each defining constraint set out in Section 3 is addressed by at least one control; the two components of the first constraint are separated by the unilateral and arms-only conditions. Bilateral whole-body movement is tested jointly by the unilateral condition, which removes bilateral symmetry, and the arms-only condition, which removes whole-body postural participation. Sensory gating is tested by the eyes-open condition, and fingertip anchoring by the movement-matched condition. Uninterrupted sensorimotor continuity is tested by the non-differentiable condition, which replaces the smooth closed path with an angular or segmented trajectory and so introduces the discrete boundaries the constraint excludes. That condition manipulates segmentation specifically; a fuller dissociation of continuity would additionally require conditions introducing pauses, reversals of direction, or an open rather than closed path, which are identified here as extensions rather than specified in the present design.

The framework predicts that removing any single constraint will measurably degrade the stability profile, but that the degradation will be qualitatively different depending on which constraint is removed. Removing bilateral symmetry through the unilateral control should primarily affect hemispheric coherence. Removing sensorimotor continuity through the non-differentiable control should primarily affect cerebellar forward model accuracy and frontal theta. Removing sensory gating through the eyes-open control should primarily affect somatosensory channel precision and cross-modal competition. Removing whole-body postural participation through the arms-only control, in which movement is restricted to the arms alone while the trunk remains stationary, should primarily affect the globally consistent multisensory stream and reduce the progressive-deepening effect. Removing fingertip anchoring through the movement-matched control, in which the movement is retained but no instruction concerning the fingertips is given, should primarily affect the measures that index attentional allocation: retrospective on-anchor report, the relationship between pacing characteristics and engagement, and interhemispheric coherence, while leaving measures attributable to movement alone comparatively intact.

The pattern of selective degradation across these five controls constitutes a systematic dissociation and is the most informative test of the mechanistic account. Two outcomes would falsify that account. If removing a constraint produces no measurable degradation, that constraint is not necessary in the manner claimed. If all the constraint-removal manipulations produce the same undifferentiated degradation, the proposal that the constraints operate through distinct mechanisms is falsified, and a single general factor — reduced sensory load, or movement as such — would provide a more economical account of the effects.

Prediction 10 — Experience-contingent pacing. When the calibration procedure is followed with no tempo instruction, cycle duration and its within-session variability are predicted to be systematically related to the attentional demand of the task, not fixed by biomechanics alone. Two tests bear on this. Pacing characteristics are modeled jointly with independently obtained engagement ratings, principally the retrospective on-anchor report, and with kinematic measures of corrective postural events. And in the movement-matched condition, where the movement is retained but no instruction concerning the fingertips is given, pacing characteristics are predicted to show a weaker and less systematic relationship with the engagement indices, since there is no attentional demand to accommodate. Absolute speed is reported empirically rather than predicted. No universal duration is predicted, and no participant is required to settle on a fixed personal tempo: pace may legitimately vary within a session as the sensory content changes. The claim is weakened if pacing shows no relation to the engagement indices, or if movement-matched pacing is indistinguishable from pacing under DPSA. The distribution of individual durations and their variability are reported as outcomes.

Prediction 11 — Relational binding versus independent division. The account developed in Section 4.1 proposes that the bilateral anchors are bound into a relational structure rather than attended as two independent foci. These alternatives make opposing predictions. If the anchors are bound in this way, three outcomes follow. A perceptual probe delivered at one anchor should be systematically affected by manipulation at the other. Any division cost should be substantially smaller than an independent-loci account predicts. And interhemispheric coherence during bilateral anchoring should exceed the level predicted from left-anchor and right-anchor unilateral conditions under a pre-specified additive null model. If instead the anchors function as two independently attended loci, probe performance at each should be separable, a measurable division cost should be present, and coherence should not exceed the level predicted by the additive null model. The latter pattern would falsify the relational-binding account developed here, and would support a divided-attention reading instead. Cross-anchor dependence alone would not establish perceptual fusion or loss of site-specific distinguishability. A control preserving bilateral stimulation while disrupting the proposed relational organization would be required to distinguish generic bilateral interaction from relational binding. Either outcome is informative. The distinction requires perceptual probing at one anchor with manipulation at the other, and is therefore tested not within the canonical unprobed protocol but as a dedicated perceptual-probe experiment, conducted in a single separate session alongside left-anchor and right-anchor unilateral comparison conditions. These two conditions belong to that dedicated experiment and are distinct from the single unilateral control specified in Section 5.2.

Together these eleven predictions make the framework empirically tractable and allow individual mechanistic components to be tested and revised independently. Each is directional, specifying not merely that a difference will be observed but what its sign and, where relevant, its relative magnitude should be. The predictions are also dissociable, since the control conditions are designed to test different mechanistic claims independently, so that falsifying one does not automatically falsify the others. And they are implementable: the primary predictions can be tested with established electrophysiological, portable neuroimaging, kinematic and autonomic methods, while some downstream predictions require dedicated perceptual or specialist imaging studies. DPSA is presented as a precisely specified, experimentally tractable framework and not as a completed theory; its claims can be evaluated, refined and, if necessary, revised through direct empirical investigation. The predictions above define what such an evaluation would involve.

## 7 Discussion

This paper has introduced Dual-Point Sensory Anchoring as a precisely specified, theoretically grounded behavioral paradigm for studying how the brain achieves and maintains attentional stability under simplified, controlled sensory conditions. The framework draws on eight theoretical traditions: predictive processing, motor control, dynamical systems, ecological psychology, embodied cognition, contemplative practice research, bilateral stimulation paradigms and large-scale brain network dynamics. Each motivates one or more of the design properties assembled here. Their partial overlap does not establish the framework; it is a reason to bring these components together within a controlled experimental paradigm. We are not aware of an existing paradigm that has assembled all of these properties simultaneously: bilateral whole-body movement, deliberate sensory gating, multi-dimensional fingertip anchoring, and uninterrupted sensorimotor continuity.

The mechanistic account developed above proposes how these four constraints may interact within the brain’s hierarchical predictive architecture to produce a low-entropy attentional regime. Prediction-error resolution is hypothesized to occur preferentially within fast, short-latency somatosensory and cerebellar circuits, producing progressive physiological stabilization that deepens across the session without deliberate top-down regulation. Sections 5 and 6 provide an initial operational specification for empirical testing and refinement, and set out eleven directional predictions, each independently falsifiable, through which the framework can be tested and revised.

Three empirical tests follow directly from the framework. The most direct is a single-session EEG study comparing the full DPSA protocol against the unilateral, non-differentiable and eyes-open conditions, with beta coherence between homologous somatosensory regions as the primary outcome measure. That design uses established mobile electrophysiological and kinematic methods, and could be implemented in any laboratory equipped for ambulatory EEG and inertial measurement unit (IMU) movement recording. A complementary priority is a longitudinal study tracking the kinematic regularity of the fingertip trajectory, beta coherence and high-frequency heart rate variability across ten to twenty sessions, testing whether DPSA effects accumulate with practice in a pattern consistent with motor skill acquisition and cortico-cerebellar model strengthening. A third priority is a hemodynamic study using portable fNIRS to test the hierarchical-confinement prediction directly, asking whether dorsolateral prefrontal hemodynamic activity decreases while accessible somatosensory and sensorimotor activity is maintained during the main anchoring phase. Cerebellar and other deep structures are not accessible to this method, so the deeper components of the account would remain untested by such a study. Conventional fMRI is incompatible with the standing movement protocol; portable fNIRS provides hemodynamic measurement within an ecologically valid context, subject to the depth limits noted above.

Beyond these immediate priorities, DPSA opens several broader research directions. The framework provides a tractable experimental instantiation in which the relationship between movement geometry and hierarchical inference can be systematically examined. Cycle duration, trajectory curvature, degree of bilateral symmetry and level of sensory gating can each be parametrically manipulated to map the precision landscape of attentional stability at a resolution existing paradigms have not permitted. The connection between DPSA and established embodied practices, including Tai Chi and Qigong, suggests a translational direction. DPSA can be understood as a mechanistic formalization of the attentional substrate common to such practices, offering a principled theoretical account of why slow, bilateral, continuous movement may promote cognitive and physiological stability. That connection invites investigations linking laboratory neuroscience with naturalistic human practice, and may support the development of accessible, low-cost attentional stabilization tools that require brief structured familiarization and little specialist equipment outside research settings.

This translational potential is particularly relevant in the context of healthy aging and cognitive resilience. Attentional stability is among the earliest cognitive capacities to show age-related decline, and the ability to engineer it through structured embodied movement, in place of pharmacological or high-demand cognitive interventions, would represent a scalable and accessible approach to cognitive health maintenance across the lifespan. The movement requires structured familiarization but no prior experience in contemplative or movement practice, and its eventual delivery may require little or no specialist equipment. In principle the paradigm is as deployable in community and home settings as in a laboratory, though the canonical research implementation includes screening, familiarization and supervision. If the core mechanistic predictions are confirmed, DPSA would provide a theoretically grounded foundation for a class of mind-body interventions whose effects on cognitive and autonomic stability can be measured and optimized. Such interventions could be adapted for older adults, for individuals with elevated attentional volatility and for communities without access to formal clinical resources. The framework therefore speaks to a broader translational challenge: developing low-burden, theoretically grounded interventions that support cognitive vitality across diverse populations and settings.

The framework has real limitations, and they are worth stating plainly. First, all predicted signatures in Section 6 are hypotheses pending systematic preregistered investigation. No empirical data are presented here, and none of the mechanistic claims have yet been directly tested. Second, no cycle duration is prescribed, and none is proposed as optimal. No pilot data, systematic or otherwise, are reported here. What is proposed is that pacing emerges from the evolving coordination regime and may vary both across participants and within a session. Whether pacing characteristics are systematically related to sensory engagement, corrective events or accumulated practice remains to be established through parametric study. Third, the framework’s claims regarding individual differences are limited to the four moderators specified below, and their characterization is a first approximation rather than a systematic mapping of boundary conditions. Fourth, the translational implications suggested here, particularly those regarding attentional volatility and accessible stabilization tools, remain speculative pending empirical confirmation of the core mechanistic predictions. Finally, the mechanistic account developed above spans multiple hierarchical levels, from peripheral tactile architecture through computational precision weighting to interoceptive and autonomic propagation. Each inferential step is theoretically grounded, but the chain as a whole has not been empirically validated end-to-end, and future work will need to establish which links in the proposed pathway are most critical and most tractable for experimental investigation.

Individual differences are treated as measured moderators, not as unexplained variance, and four are specified in advance. Baseline somatosensory precision is characterized before the first session by two-point discrimination and monofilament detection thresholds at the index and middle fingertips, on the reasoning that the framework’s central claim concerns precision-weighting of the tactile channel and that its empirical test should therefore be conditioned on how precise that channel is at baseline. Attentional capacity is characterized by a standard sustained-attention measure administered at enrolment. Motor learning rate is characterized by the change in cycle-duration variability across the familiarization sessions, a characterization that requires no additional testing because it is derived from the calibration data already collected. Autonomic reactivity is characterized by resting HF-HRV and RMSSD recorded before the first session.

These four are entered as covariates in the primary analyses, and the framework makes two directional predictions about them. Aerobic glycolysis in task-relevant tissue scales with individual differences in motor adaptation rate (Shannon et al., 2016), which suggests that the rate at which the coordination regime is acquired should predict the magnitude of the stabilization effects rather than being independent of them. Participants with faster acquisition are therefore predicted to show earlier convergence of cycle-duration variability and larger session-to-session gains, while baseline somatosensory precision is predicted to moderate the size of the anchoring effect specifically, not the effects attributed to movement or sensory gating. Should the moderators show no relationship to the outcomes, the framework would not be falsified; its account of which individual characteristics matter would require revision. Where variance remains substantial after these covariates are included, it is reported rather than absorbed, since the boundary conditions of the paradigm are themselves an object of investigation.

The limitations set out above are those of a framework at the start of its empirical life. DPSA is offered not as a finished theory but as a specified and falsifiable account of how the design of bilateral sensory input — its geometry, temporal organization, postural grounding and attentional architecture — shapes the conditions under which attentional stability arises. The most informative initial test is the single-session comparison of the full protocol against the unilateral, non-differentiable and eyes-open conditions, with interhemispheric beta coherence as the primary outcome. If that comparison shows no differentiation, the proposed bilateral-interaction and relational-binding mechanism would require substantial revision.

## Data Availability

The original contributions presented in the study are included in the article, further inquiries can be directed to the corresponding author.
